# Case of Nocturnal Emesis, Weight Loss, and Aspiration Pneumonia

**DOI:** 10.1177/00099228231171065

**Published:** 2023-04-29

**Authors:** Roman Babayev, Sharonda Taylor, Elizabeth V. Franklin

**Affiliations:** 1 Department of Adolescent Medicine, Baylor College of Medicine, Texas Children’s Hospital, Houston, TX, USA

**Educational Objectives**

- Increase awareness of eating disorders and their medical complexities, such as achalasia.
- To explore the relationship of eating disorders with intentional emesis as a risk factor for achalasia.

## Case Report

MR, age 11 years, assigned female at birth who presented to the emergency department (ED) with a chief complaint of weight loss. She lost 19% of her body weight in the previous 4 months. MR’s father reported she had intentionally restricted diet and vomited for the past 8 months prior to alter her body shape and weight. He caught her purging multiple times but it eventually became unintentional, along with a gradual intolerance of solids.

Two weeks before visiting the ED, she was hospitalized at an inpatient psychiatric unit for 2 days and began sertraline for depression and suicidal ideation. MR had no history of mental health treatment or psychiatric medication use prior to this admission. Upon discharge from that facility, the family was advised to seek care for an eating disorder due to purging and restrictive eating behaviors.

MR also had a cough with copious oral and nasal secretions. Her primary care physician (PCP) had prescribed her amoxicillin a month prior to being admitted to the psychiatric facility. The cough had not resolved. After leaving the psychiatric facility, she was prescribed amoxicillin clavulanate by her PCP for presumed pneumonia due crackles on examination but did not start it.

During her physical examination in the ED, MR was alert, awake, and appeared anxious as she made limited eye contact. Her vital signs were blood pressure 129/63, pulse 132, oral temperature 37.1°C, respiratory rate 24, and intermittent hypoxia (oxygen saturation 82%). Anthropometrically, she had weight 35.2 kg (25%), height 156.5 cm (94%), and a body mass index (BMI) 13.9 kg/m^2^(1.88%). The orthostatic vitals were consistent with postural orthostatic tachycardia syndrome (POTS), manifesting as a pulse increase at 5 minutes of 50 bpm. Pertinent physical exam findings include thin appearance with bony prominence of the iliac and thoracic regions, posterior pharyngeal erythema with no purulence nor adenopathy, and mild crackles on the right lower lobe. Cardiac examination was normal. Comprehensive metabolic panel, complete blood count and thyroid levels were obtained and notable for abnormalities of white blood cell count of 12,000 × 10^3^ uL, thyroxine 14.6, thyroid stimulating hormone 1.7 uIU/ml, and CO_2_ 29 mMol/L. The remaining laboratories were normal including inflammatory markers.

**Figure 1.**
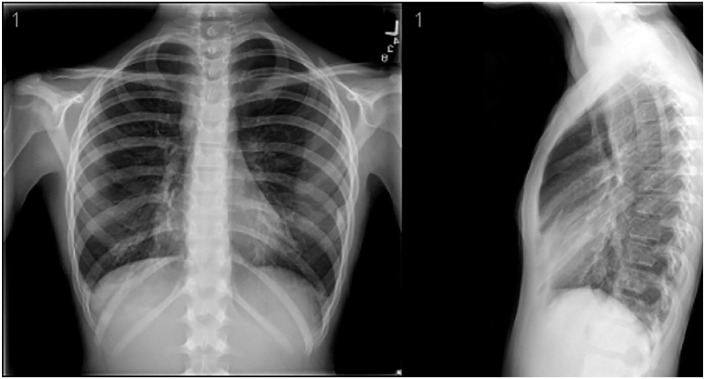
Chest x-ray with right lower lobe infiltrate.

An electrocardiogram showed normal sinus rhythm. A chest x-ray was also completed (see Figure 1).

Initial diagnoses of anorexia nervosa binge-purge subtype, moderate malnutrition, and aspiration pneumonia were made, and MR was admitted to the hospital.

## Hospital Course

At admission, MR was estimated to be 77.5% of treatment goal weight (normed to the 50th percentile for BMI), with POTS, risk for refeeding syndrome, presyncope, constipation, and aspiration pneumonia.

MR was placed in a video-monitored room and started on a 40 kcal/kg/day diet. She received oral PhosNaK for the first 5 days to prevent refeeding syndrome, as per protocol. During the first week, she was trialed on lorazepam and then hydroxyzine and ondansetron prior to meals, but she continued to have intermittent involuntary emesis. Though she had initially restricted her intake and purged to lose weight, she now stated that she wanted to eat and gain weight but was refusing food due to a fear of unintentional vomiting.

A psychologist was consulted per the eating disorder treatment protocol as well as for identification of additional psychosomatic causes of the involuntary emesis, and for coping with the fear of vomiting. During the diagnostic intake, MR and her father reported multiple symptoms of anorexia nervosa—binge/purge subtype, including intentional restriction of her food intake to change the shape and size of her body, intense fear of weight gain, daily episodes of binging and purging, and distorted body image (e.g., thoughts related to feeling she is “fat” despite low body weight). Most notably, MR said that while she was intentionally vomiting for many months after having daily episodes of binging, she has not been intentionally vomiting the past few months and felt that no matter what she was eating or drinking she would vomit. She described attempting to restrict her intake the past few weeks prior to this admission in the hopes that it would result in reduced unintentional vomiting but stated food restriction did not seem to impact whether or not she vomited.

Her symptom onset coincided with several family and personal stressors, including her mother’s physical absence in another state throughout her entire admission, two younger siblings with special healthcare needs, a history of significant body shaming by peers, and gender dysphoria. At intake, MR also met criteria for Generalized Anxiety Disorder. Her father described significant stress related to working full time and managing three young children while his wife was out of the state. While MR appeared to be comforted by her father, she also expressed guilt and sadness related to feeling that she is adding burden to her family by requiring a hospitalization.

Nurses reported that some of the emesis episodes took place during the night, while the patient was sleeping, and while some episodes were minimal, others were more significant. A nasogastric tube was placed, and MR was started on erythromycin for suspected delayed gastric emptying. A gastroenterologist was consulted, and upon further questioning, the patient stated that she typically did not have abdominal pain or nausea but upon taking food PO, she would passively vomit and spit out undigested food a few minutes after intake. The patient denied dysphagia or a sensation of food getting stuck. The gastroenterology (GI) service recommended diaphragmatic breathing for anxiety and famotidine and polyethylene glycol to prevent possible rumination and reflux, which showed no benefit. *Helicobacter pylori* antigen and celiac panels were negative.

**Figure 2.**
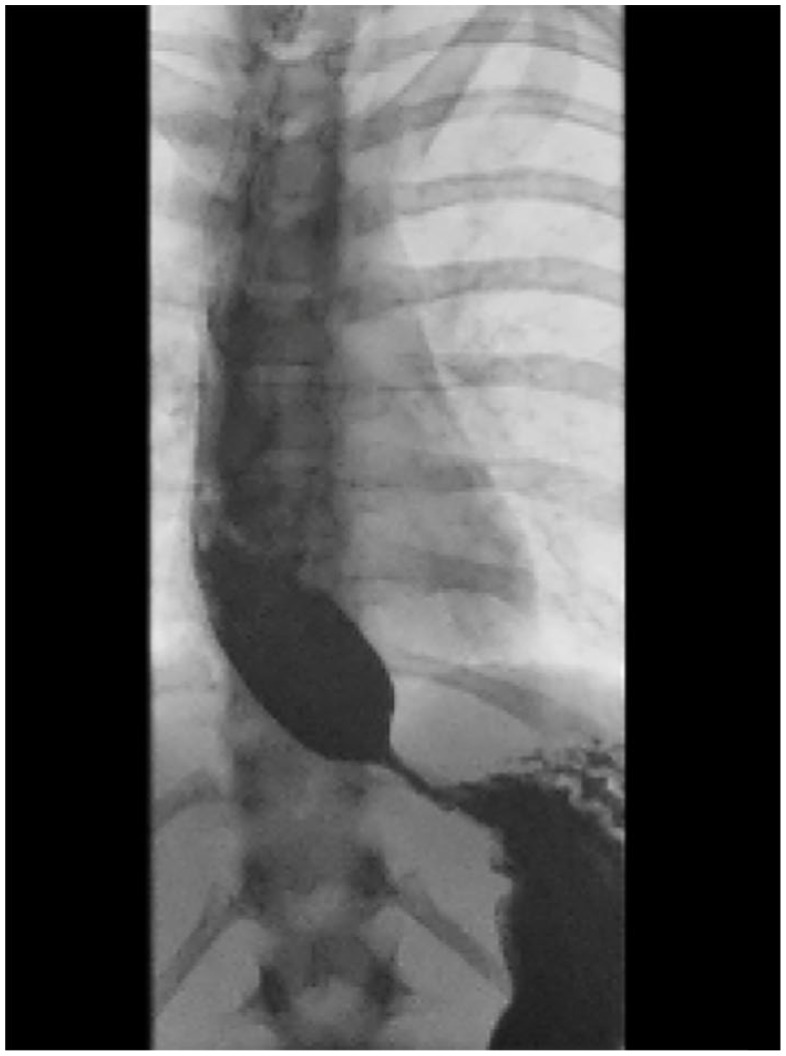
Chest x-ray, barium swallow study showing bird beak sign.

Despite teaching MR both cognitive and behavioral relaxation strategies for managing urges to vomit or fear of vomiting, after another week of no improvement of emesis, the GI team was again consulted for further evaluation of possible esophageal web or diverticulum. They conducted a barium swallow study which showed a bird beak sign (see Figure 2), and manometry showed pan-esophageal pressurization and impaired lower esophageal sphincter relaxation consistent with type II achalasia. An outpatient Heller myotomy and fundoplication with surgery were scheduled and MR continued to receive nutritional rehabilitation via the NG tube that was distal to the achalasia. She was discharged from the hospital in December and continued to receive outpatient individual and family therapy to manage anxiety related to vomiting after meals until she underwent surgery. Almost immediately upon receiving the surgery, MR no longer endorsed a fear of vomiting and was able to eat without concerns.

## Discussion

Achalasia is defined as diminished relaxation of the lower esophageal sphincter and the absence of peristalsis. It is most often idiopathic but can be related to autoimmune processes such as sarcoidosis, infectious processes such as Chagas’s disease, and malignancy. The most common symptoms are emesis, regurgitation, dysphagia, and weight loss. A definitive diagnosis is made with a barium swallow study and esophageal manometry.

As per the American Society of Gastrointestinal Endoscopy achalasia is a primary esophageal motor disorder of unknown etiology characterized by degeneration of the myenteric plexus, which results in impaired relaxation of the esophagogastric junction, along with the loss of organized peristalsis in the esophageal body.^1^ Many proposed triggers have been suggested for the destruction of the inhibitory neurons of the myenteric plexus, but the exact mechanism of the damage remains unclear. The standard of care for achalasia is Heller myotomy and fundoscopy with a predicted probability of improvement in dysphagia has been reported to be 91% at 12 months. The annual incidence of achalasia in childhood is 0.11/100,000.^2^ The causal relationship between an eating disorder and achalasia remains unclear. A literature review identified 36 cases of achalasia which were misdiagnosed and mismanaged as an eating disorder. Five case reports described diagnostic comorbidity between achalasia and bulimia nervosa,^3^ leading the authors to question whether chronic induced emesis can lead to achalasia. Some case reports suspect achalasia as a complication of bulimia nervosa.^4^

In our case report, our patient exhibited clear symptoms of an eating disorder, in that she was initially restricting food and purging to avoid weight gain and to lose weight. She also reported poor body image and body discomfort during her initial months of restriction and intentional purging. However, over time, she noted that the emesis became unintentional and that she was unable to keep food down despite a desire to do so. It is possible that her history of chronic emesis due to an eating disorder predisposed her to physical damage of the myenteric plexus, leading to the achalasia. MR’s complex psychiatric history and current psychosocial environment made it challenging at times to differentiate physical and psychological triggers of the emesis episodes. We believe that other patients like MR may not be initially identified as having achalasia due to a long history of self-reported induced purging behaviors and comorbid psychiatric conditions. While achalasia in childhood is rare, this case report highlights the need to further evaluate patients presenting with involuntary emesis, especially if the vomiting is occurring at night and regardless of the size of the meal.

Critically, once MR underwent surgery, she was able to eat by mouth without vomiting and no longer experienced anxiety related to eating. This case study suggests that a history of an eating disorder, especially with chronic emesis, may place some children at risk for developing achalasia.

## Final Diagnosis

Our patient was treated for anorexia nervosa—binge/purge subtype because she met the criteria for severe malnutrition with significant fear of weight gain with initial intentional emesis multiple times per week for several months. She also met criteria for generalized anxiety disorder as per *DSM*-V (5th ed.; *DSM*–5),^5^ and achalasia. She received nasogastric tube feeds on an inpatient basis and was discharged after achieving 85% of her ideal body weight. In addition to replenishing her nutrition, we used a multidisciplinary approach, including having her meet regularly with a physical therapist, registered dieticians, and regularly with an individual and family psychologist. Following a Heller myotomy and fundoplication for achalasia outpatient, she was able to successfully eat by mouth and no longer required outpatient therapy.

## Conclusion and Lessons Learned

This case report offers insight into and increases awareness of the likelihood of developing achalasia from repeated intentional emesis. When a patient presents with an eating disorder with emesis, considering achalasia is generally low on the differential diagnosis list. But while relatively uncommon, achalasia can be a complication of chronic emesis among patients with eating disorders with purging behavior, although it may be unclear if it is a result of the eating disorder or a separate diagnosis. A detailed history and thorough physical exam can help a clinician rule out other organic causes of passive emesis, especially when sleeping, such as superior mesenteric syndrome or esophageal web stricter. Other differentials diagnoses should also be considered, such as somatization from anxiety.

## Author Contributions

RB: Contributed to conception and design, Drafted the manuscript, Gave final approval, Agrees to be accountable for all aspects of work ensuring integrity and accuracy. ST: Contributed to conception and design, Gave final approval, Agrees to be accountable for all aspects of work ensuring integrity and accuracy. EVF: Contributed to conception and design, Gave final approval, Agrees to be accountable for all aspects of work ensuring integrity and accuracy.
